# Pulse oximeter bench tests under different simulated skin tones

**DOI:** 10.1007/s11517-024-03091-2

**Published:** 2024-04-24

**Authors:** Suvvi K. Narayana Swamy, Chenyang He, Barrie R. Hayes-Gill, Daniel J. Clark, Sarah Green, Stephen P. Morgan

**Affiliations:** 1 Optics and Photonics Research Group and Centre for Healthcare Technologies, University of Nottingham, University Park, Nottingham, UK; 2 Clinical Engineering Department, Nottingham University Hospitals NHS Trust, Nottingham, UK

**Keywords:** Pulse oximeter, Oxygen saturation, Transmission-mode, Occult hypoxemia, Racial bias, Melanin, Skin colour

## Abstract

Pulse oximeters’ (POs) varying performance based on skin tones has been highly publicised. Compared to arterial blood gas analysis, POs tend to overestimate oxygen saturation (SpO_2_) values for people with darker skin (occult hypoxemia). The objective is to develop a test bench for assessing commercial home and hospital-based POs in controlled laboratory conditions. A laboratory simulator was used to mimic different SpO_2_ values (~ 70 to 100%). Different neutral density and synthetic melanin filters were used to reproduce low signal and varying melanin attenuation levels. Six devices consisting of commercial home (Biolight, *N* = 13; ChoiceMMed, *N* = 18; MedLinket, *N* = 9) and hospital-based (Masimo Radical 7 with Neo L, *N* = 1; GE B450 Masimo SET with LNCS Neo L, *N* = 1; Nonin 9550 Onyx II™, *N* = 1) POs were reviewed and their response documented. Significant variations were observed in the recorded SpO_2_ values among different POs when exposed to identical simulated signals. Differences were greatest for lower SpO_2_ (< 80%) where empirical data is limited. All PO responses under low signal and melanin attenuation did not change across various simulated SpO_2_ values. The bench tests do not provide conclusive evidence that melanin does not affect in vivo SpO_2_ measurements. Research in the areas of instrument calibration, theory and design needs to be further developed.

**Graphical Abstract:**

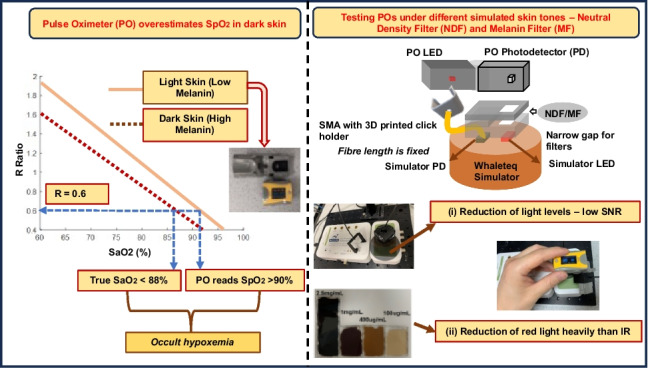

**Supplementary Information:**

The online version contains supplementary material available at 10.1007/s11517-024-03091-2.

## Introduction

Since the outbreak of COVID-19, over 6.9 million people have died from this disease globally [1]. Silent hypoxia, also known as ‘happy hypoxia’, is a common symptom in patients infected with this virus. It is defined as a condition during which the patient experiences no dyspnea or respiratory illness but suffers a sudden decrease in blood oxygen levels [2]. If undetected it may cause pneumonia and rapid damage to internal organs [2]. Therefore, in high-risk patients, continuous monitoring of blood oxygen saturation is recommended to facilitate timely problem detection and delivery of medical treatment and care.

Arterial blood gas (ABG) sampling is considered the gold standard for measuring arterial oxygen saturation (SaO_2_), traditionally performed by a blood gas analyzer. However, this approach is impractical for routine or home-monitoring as it is invasive, painful, intermittent and time-consuming [3]. Alternatively, a pulse oximeter (PO) is commonly used as it allows for non-invasive, painless, continuous and inexpensive monitoring of peripheral oxygen saturation (SpO_2_) in real-time. Pulse oximetry traditionally uses red (660 nm) and infra-red (IR, 880 nm or 940 nm) light and utilises photoplethysmography (PPG) which records the absorption of light due to blood volume variations. A PPG signal consists of two main components: AC which is a pulsatile signal synchronous to the cardiac cycle; and DC which refers to light from non-pulsatile components [4].

In the United Kingdom, the COVID Oximetry @home programme was launched as part of the National Health Service (NHS) response to the pandemic. Through this program, commercially available peripheral Pulse oximeters (PO)s (transmission-mode) were delivered to allow patients with COVID-19 or at high risk to remotely monitor their SpO_2_ at home [5]. These devices are widely used globally by healthcare professionals to make timely clinical decisions when admitting patients, particularly with the absence of any noticeable symptoms to hospitals for critical care and treatment. However, several recent retrospective clinical studies have highlighted that SpO_2_ may be overestimated [6–10] in patients with non-white skin types. This phenomenon is generally termed occult hypoxemia where SpO_2_ measured by a PO is greater than 90%, despite SaO_2_ being less than 88% [6]. For example, one large-scale retrospective study by Sjoding et al. concluded that the rate of occult hypoxemia was three times higher in black patients than in white patients [6]. Crooks et al. observed a similar SpO_2_ but a decrease in SaO_2_ and an increase in breathing rate in non-white patients compared to white patients at the time of transfer to the intensive care unit [11]. SpO_2_ overestimation related to skin pigmentation concerning the accuracy of POs has also been observed in earlier studies [12–14], all suggesting that when SpO_2_ is measured by a PO, patients with dark skin have a higher risk of undetected hypoxemia and therefore delayed clinical treatment relative to white-skinned patients.

Previous studies have compared pulse oximetry with ABG during clinical care. However, these comparisons have potential sources of error due to variability in local experimental protocol and skin colour differences between patients. It is therefore valuable to construct a test bench to evaluate a range of POs under different controlled experimental conditions such as SpO_2_ levels, light levels and skin colours. Dark skin has high melanin content that attenuates more DC light, and also preferentially absorbs more red light than IR. High melanin concentration in skin has two main effects (i) reduction of overall detected light levels (resulting in low signal-to-noise ratio) [15]; (ii) attenuation of red light more heavily than IR [16]. As a result, it is important to test these POs commonly used at home and in hospitals whilst mimicking the two principal effects of melanin in the skin.

To this end, a laboratory benchtop simulator has been developed in which different signals can be presented to POs to mimic typical physiological signals in a controlled and standardised way. This has primarily been focused on simulating different SpO_2_ levels; and different signal amplitudes to reproduce the effects of low signal-to-noise ratio and different melanin concentrations on device performance. The advantage of this method is that it offers a simple approach to evaluating POs by avoiding the need for more complex and invasive ABG analysis, which would be necessary in studies involving individuals with different skin tones. The main aim of this study is to evaluate the performance of the range of POs used in the NHS COVID Oximetry @home programme under the effects of low signal and varying melanin attenuation levels across a range of simulated oxygen saturation values.

## Methods

### Background

The basis of pulse oximetry is the ‘ratio of ratios’ (*R*) obtained from the PPG [17].

1 $$R={~}^{\frac{{AC}_{red}}{{DC}_{red}}}\!\left/ \!{~}_{\frac{{AC}_{IR}}{{DC}_{IR}}}\right.$$

where AC is the amplitude of the detected signal from the pulsatile blood and DC is the detected signal level due to static tissue, measured at red and IR wavelengths.

**Fig. 1:**
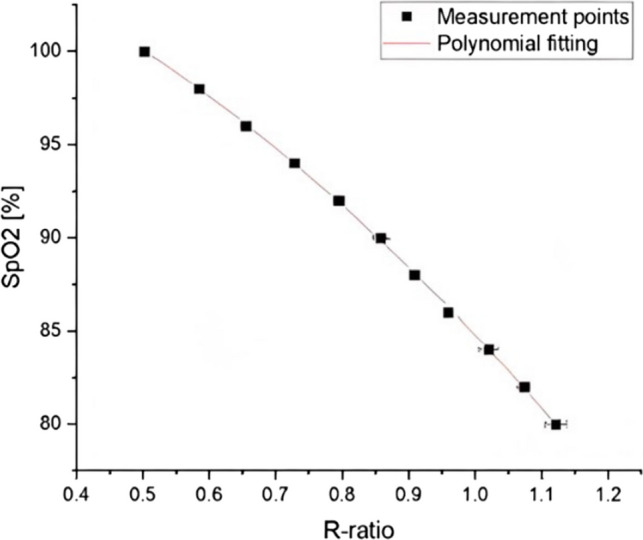
Example of the relationship between SpO_2_ and the *R*-ratio

Measurement of R helps to compensate for differences in light source intensity and, in theory, skin colour as these effects cancel as they are present in the numerators and denominators in Eq. (1). Due to the different light scattering properties of tissue at red and IR wavelengths, measurements of *R* are calibrated against ABG to provide an empirical inverse relationship between *R* and SpO_2_. An example of this relationship is shown in Fig. 1 (figure based on data from [18]). Different manufacturers have different R-curves (caused by slight changes in the geometry and wavelengths) which are proprietary and are not publicly available.

### Devices under test

A list of transmission mode POs deployed for bench testing can be found in Table 1. POs (1–3) namely Biolight, ChoiceMMed and MedLinket were provided by NHS @home and the numbers depended on stock availability at the time. ChoiceMMed had four distinct display models, for easy identification, were internally labelled as ChoiceMMed A, B, C and D. POs 4) to 6) are examples of those used routinely at Nottingham University Hospitals NHS Trust. Finally, POs 4) and 5) were both tested with the Masimo low noise cabled sensors (LNCS) Neo L Sensor. Photographs of all the sensors are shown in Appendix 1. The central wavelength that these POs operate are at 660 nm and 880 nm with the exception of the Masimo LNCS sensor, which uses 940 nm.

**Table 1:** A list of test devices deployed for the bench tests along with the quantity of each tested

Name of the brand	Quantity	Manufacturer	Model
1. Biolight	13	Guangdong Biolight Meditech Co., Ltd	M70
2. ChoiceMMed i. A ii. B iii. C iv. D	18 4 2 6 6	Beijing Choice Electronic Technology Co., Ltd	MD300C21
3. MedLinket	9	Shenzhen Med-link Electronics Tech Co., Ltd	AM-801
4. Masimo Radical-7 with LNCS Neo L	1	Masimo Corporation	Radical-7
5. GE B450 Masimo SET with LNCS Neo L	1	GE Healthcare Finland Oy	B450-01
6. Nonin 9550 Onyx II™	1	Nonin Medical, Inc	9560BT

### Test bench

**Fig. 2:**
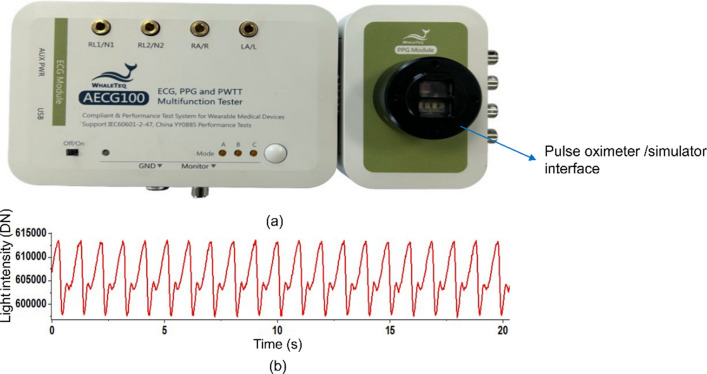
**a** PPG simulator (AECG100, Whaleteq, Taiwan). The interface is configured for reflection geometry as the light source and detector are on the same side of the device. **b** An example of the simulated signal. AC ~ 15,000 ‘light units’ (DN)s sitting on a DC level ~ 605,000 (DN)s

The core of the test bench is a PPG simulator (AECG100, Whaleteq, Taiwan) to set the intensity of red and IR light entering the PO (Fig. 2a). The DC light intensity could be set within a range from 100 mV to 3000 mV and was maintained at 2000 mV for both red and IR light. Meanwhile, the AC light intensity varied between 0.75 mV to 30 mV. In order to generate PPG signals corresponding to different saturation levels, the IR AC intensity was fixed at 20 mV while the red AC intensity was varied across different voltages. These adjustments control the *R*-ratio which is used to calculate SpO_2_ (Eq. (1)). The pre-set AC and DC voltage levels for red and IR light corresponding to three different *R*-ratios are displayed in Table 2.

**Table 2:** The preset AC and DC voltages for red and IR light that correspond to three different *R* ratios

*R* ratio	Red AC (mv)	Red DC (mv)	IR AC (mv)	IR DC (mv)
0.65	13	2000	20	2000
1	20	2000	20	2000
1.4	28	2000	20	2000

The interface between the PO and the simulator comprises red (660 nm) and IR (880 nm or 940 nm) light sources to send controlled optical signals to the PO and a photodetector to receive light from the PO. The photodetector on the simulator enables timing synchronisation between PO and the simulator. The simulator is designed for evaluating reflection geometry POs and so the light sources and detector are situated on the same side of the device. Although, the test devices operated under two different IR light sources (880 nm or 940 nm), the test could be carried at either wavelength by changing the source of the Whaleteq.

**Fig. 3:**
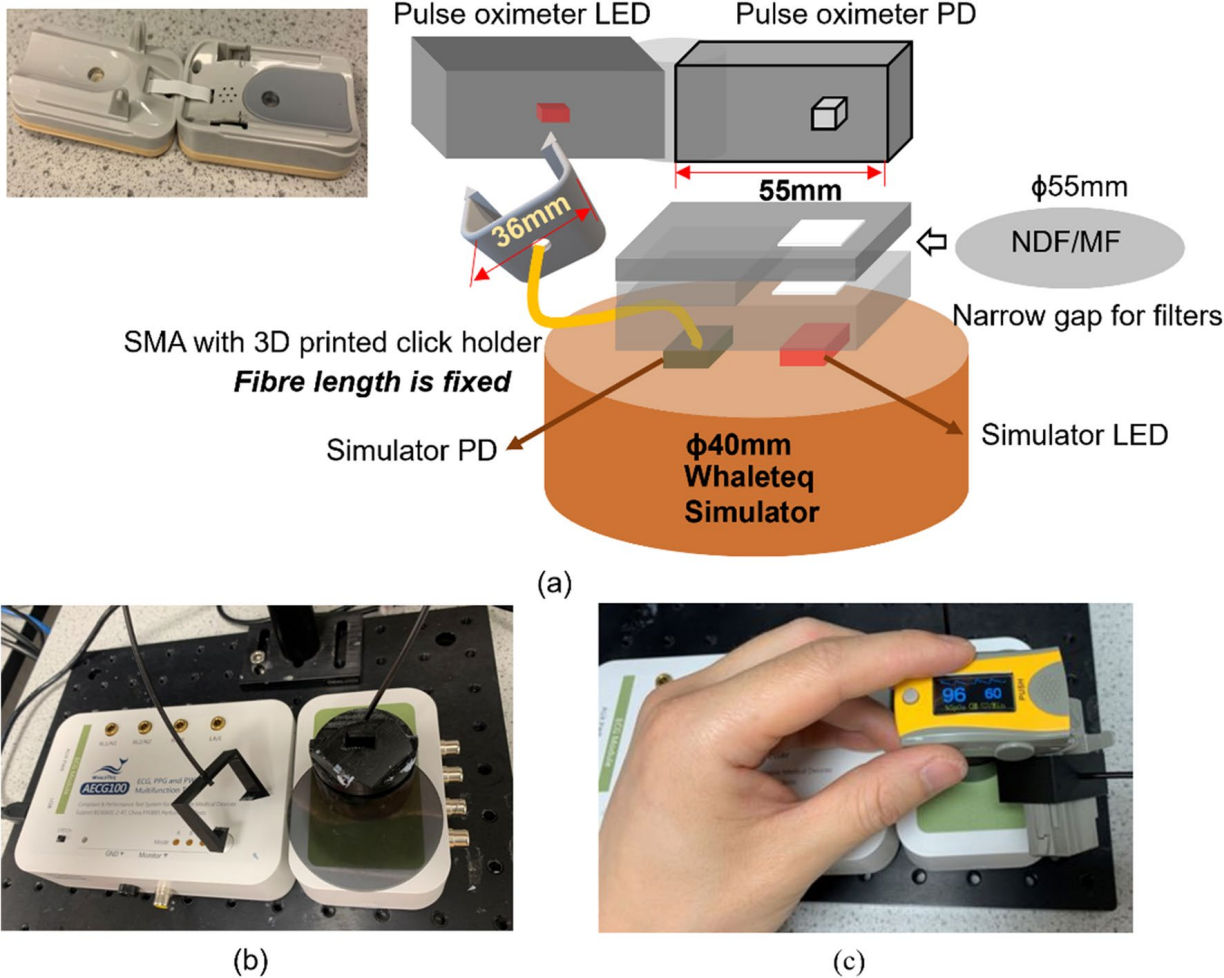
Test bench **a** the photodetector (PD) of a disassembled PO is positioned directly over the Whaleteq simulator light sources. Light from the PO is routed to the simulator detector via an optical fibre. The 3D-printed holder allows the filter to be positioned between the simulator and the PO. (LED—light emitting diode, PD—photodetector, PO—pulse oximeter). **b** photograph of the test bench including a neutral density filter attenuator. **c** with disassembled PO under test

To allow the Whaleteq simulator to be used with transmission geometry (finger-clip) PO, it was necessary to disassemble each PO so that they could be laid flat. For each PO, a 3D-printed holder was constructed and mounted on top of the simulator. Figure 3 shows a diagram of the test bench in which the PO’s photodetector is positioned directly above the light sources of the simulator. The light source from the PO is routed to the photodetector of the simulator via a fibre optic cable. The 3D printed holder includes a barrier to prevent direct coupling of light between the source and the detector. A gap in the 3D-printed holder is included to allow filters (neutral density or melanin filters) to be inserted to attenuate the light presented to the PO.

#### Effect of neutral density filters

**Fig. 4:**
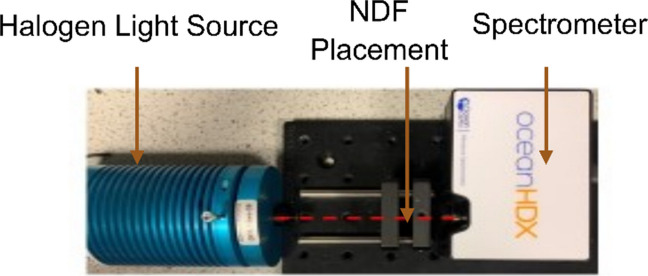
NDF attenuation versus wavelength measurement system showing halogen lamp (HL2000, Ocean Optics) with a spectrometer (HDX, Ocean Optics)

To investigate the performance of each PO at low signal intensities, neutral density filters (NDF)s were used to attenuate the light equally at red and IR wavelengths. Although this theoretically could be achieved by reducing the voltage across the light source on the simulator, in reality, the relationship between voltage and optical intensity is non-linear which would have made interpretation more complicated. This is overcome by maintaining a fixed DC light level from the simulator and changing the intensity presented to each PO by an NDF. Figure 4 shows the setup used to confirm the NDF attenuation as a function of wavelength including a halogen light source (HL 2000, Ocean Optics) and a spectrometer (HDX, Ocean Optics).

#### Effects of synthetic melanin filters

**Fig. 5:**
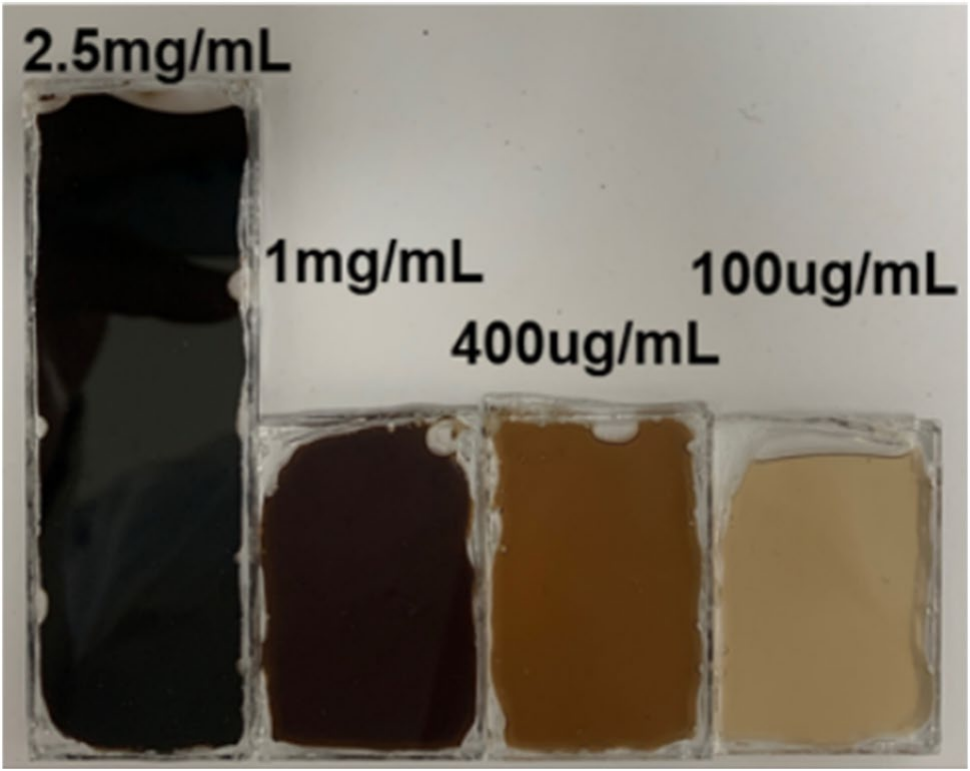
Synthetic MFs with four different melanin concentrations

The effect of skin colour is mimicked by manufacturing synthetic melanin filters (MF)s from different concentrations of synthetic melanin (M8631, Sigma-Aldrich). These attenuate red and IR wavelengths by different amounts (red attenuation greater than IR). Four slides were fabricated to cover 4 bands that simulated white to very dark skin as shown in Fig. 5 and were stratified based on both subjective and objective skin colour measurement methods. The subjective evaluation utilised the Monk Skin Tone Scale [19], a 10-shade scale (Letters A–J) representing human skin colour. Objective measurement involved determining CIE Lab* values and Individual Typology (ITA) angle [20, 21], from the transmitted spectrum shown in Fig. 5. Table 3 summarises the characterisation of the melanin slides using both the Monk Skin Tone Scale and the CIE Lab* colour space technique. Appendix 2 outlines the steps for quantitatively calculating colour information from the four transmitted spectra of melanin slides.

**Table 3:** The characterisation of the melanin slides based on Monk Skin Tone Scale and CIE Lab* colour space technique

Filters	Monk Skin Tone Scale	L*	a*	b*	ITA°	Skin colour
100 μg/ml	D	86.44	2.28	11.53	72.43	Very light
400 μg/ml	F	64.84	11.32	25.13	30.56	Intermediate
1 mg/ml	I	34.36	22.25	11.34	− 54.46	Dark
2.5 mg/ml	J	13.23	17.71	-13.98	69.17	Very light

It should be noted that the 2.5 mg/ml melanin filter ought to be classified as ‘dark’, as it is darker than the 1 mg/mL sample, evidenced by its L* value, which indicates lightness (where a lower L* signifies a darker color), and its categorisation on the Monk Scale. Despite this, the ITA angle and indicated skin color do not accurately reflect this difference. This discrepancy is likely due to the low signal-to-noise ratio, as observed in the low values in the transmission spectrum for the 2.5 mg/mL sample.

## Results

Initially, the POs were tested across a range of simulated SpO_2_ (varying *R* values) (Sect. 3.1). This was followed by introducing NDFs to reduce the signal-to-noise ratio while affecting red and IR wavelengths equally (Sect. 3.2). Finally, MFs were used to attenuate light by different amounts (Sect. 3.3).

### Varying SpO_2_, no attenuation filters

**Fig. 6:**
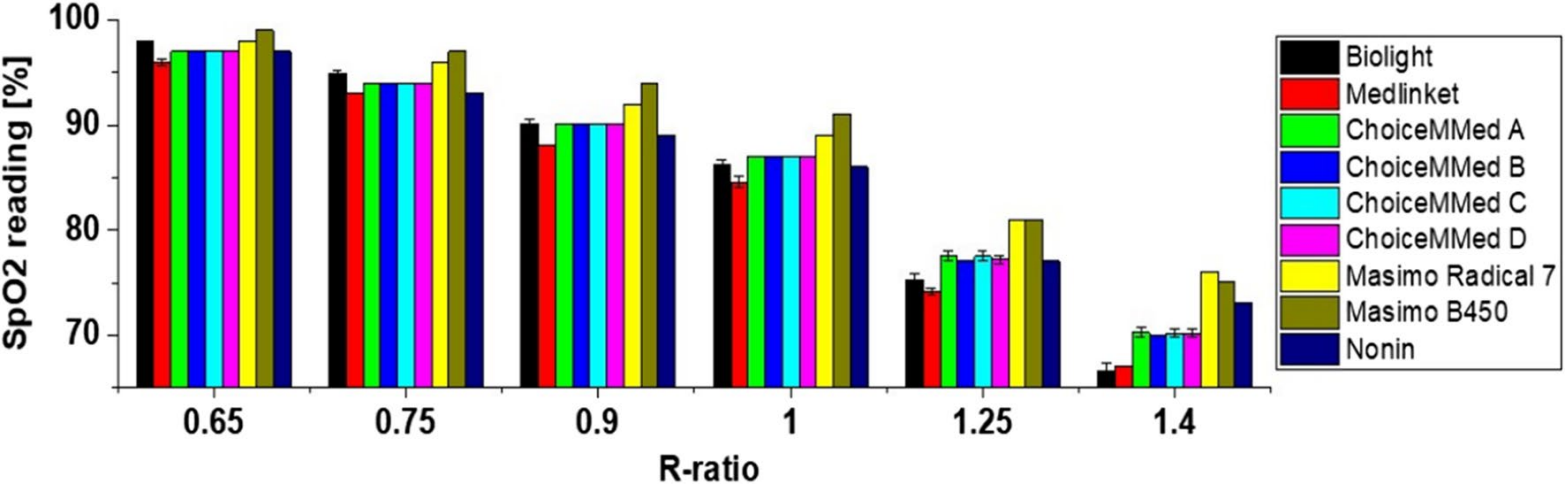
SpO_2_ values recorded by different POs for a range of R-values generated by the Whaleteq simulator

Initially, the devices were tested without introducing any additional attenuation at different *R*-ratios: 0.65, 0.75, 0.9, 1, 1.25, 1.4. This corresponds to a typical SpO_2_ value of > 95% (*R* = 0.65) to 60–70% (*R* = 1.4). Figure 6 shows SpO_2_ values recorded by different POs for a range of different *R* values. The horizontal axis shows the controlled change of the *R*-ratio using the PPG simulator. Each of the bars represents the SpO_2_ value recorded by each PO. As the *R*-ratio increases the SpO_2_ value from each device decreases—this is consistent with the empirical relationship between *R* and SpO_2_ (Fig. 1). Within each device the error was relatively low (< ± 1% absolute) in all cases (seen from the standard deviation of each SpO_2_ value in Fig. 6—shown as small error bars).

In some cases, the number of devices tested was small (Table 1) so errors of 0% (i.e., no error bar) should be treated with caution. Masimo devices typically recorded higher values of SpO_2_ than other devices. The greatest differences between devices can be observed at high *R*/low SpO_2_ values, e.g., for *R* = 0.65 the different devices display 96–98%, whereas at *R* = 1.4 the devices display 66–76%. This is possibly due to the paucity of human subject calibration data available to manufacturers in the low SpO_2_ range. ChoiceMMed versions A–D record very similar values for all device types. Although they are physically different (Appendix 1) it is likely just due to a different display rather than any fundamental difference in the underlying technology. Therefore, for further tests (Sects. 3.2, 3.3) only one version of the ChoiceMMed devices was used.

### Varying SpO_2_, with neutral density filters

**Fig. 7:**
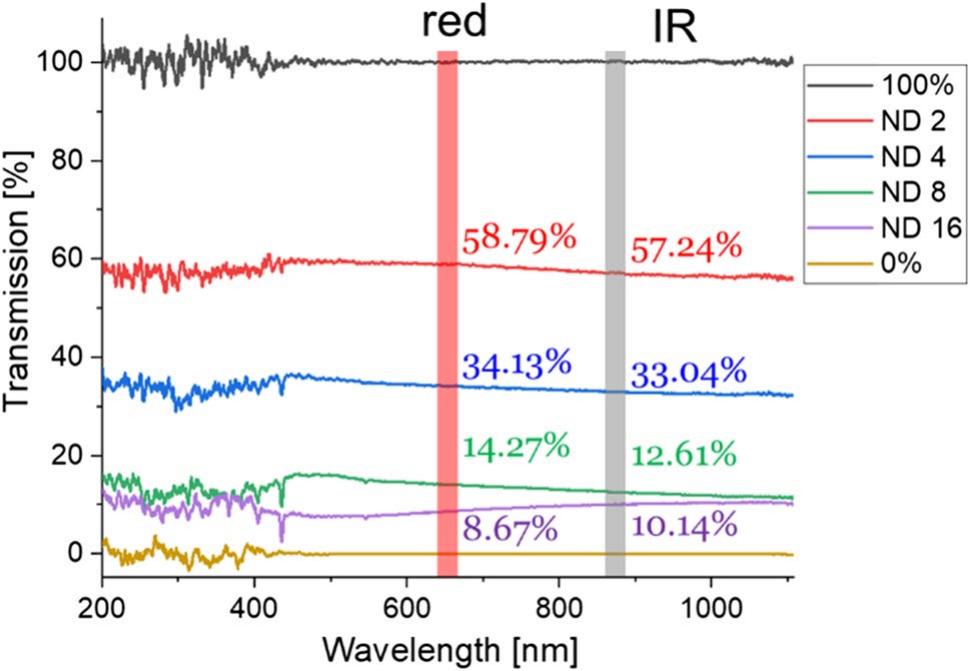
The measured NDF attenuation spectrums show approximately equal attenuation of red and IR wavelengths for all NDF orders

The NDFs were used to attenuate red and IR light equally and hence it was possible to simulate the effects of low signal levels separately from the attenuation by melanin. The measured transmission spectra (Fig. 7) show approximately equal attenuation at red and IR wavelengths for different NDFs. Transmission values at wavelengths < 400 nm are noisy as the spectral intensity of the halogen lamp is low in this range.

**Fig. 8:**
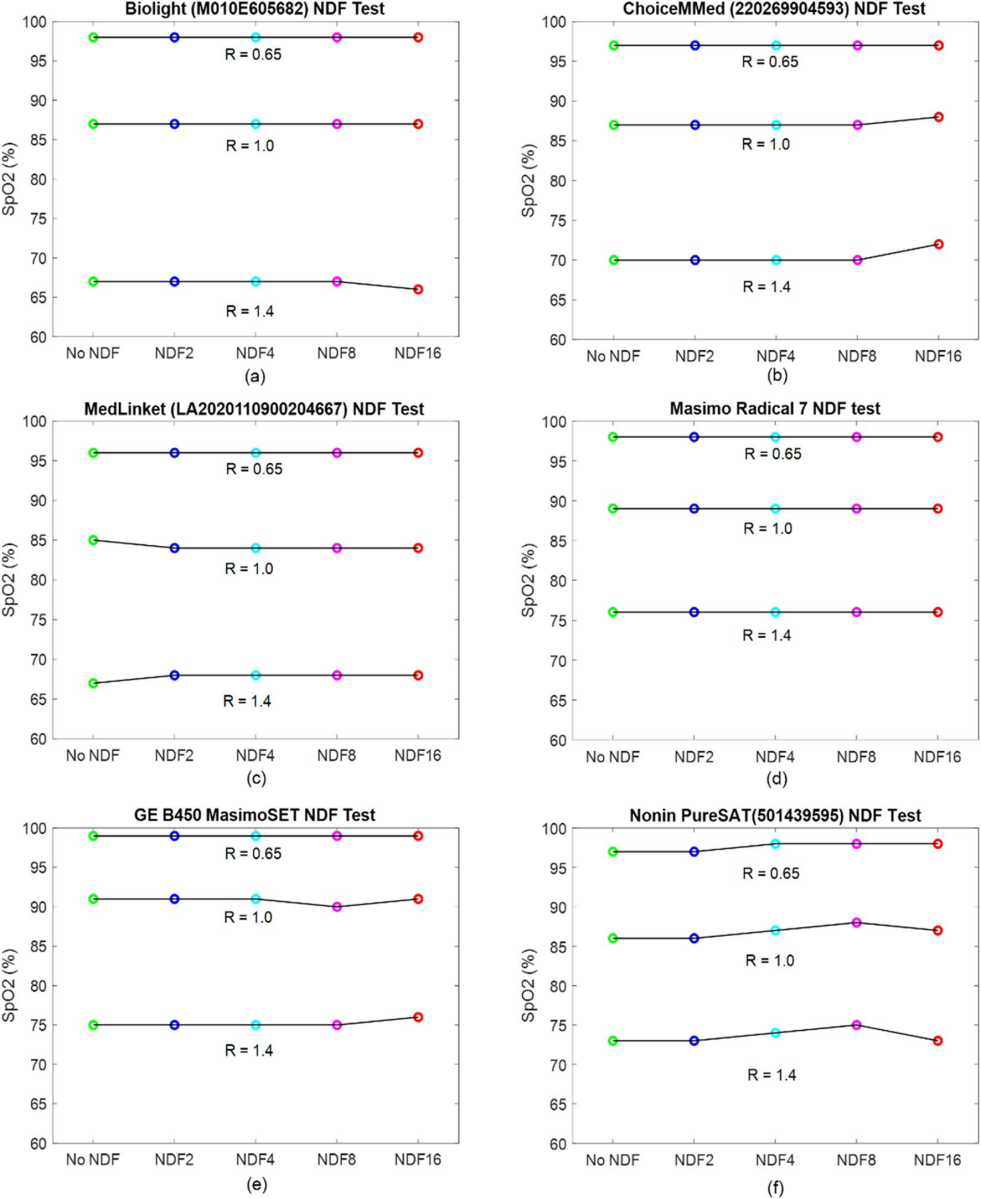
Effect of NDF filters attenuating red and IR light equally as a function of R generated by the Whaleteq. NDF2 = ½ light transmitted, NDF4 = ¼ etc. for different PO devices. **a** Biolight; **b** ChoiceMMed; **c** MedLinket; **d** Masimo Radical 7; **e** Masimo SET; **f** Nonin PureSAT

The response of each PO under different NDFs has been recorded and is shown in Fig. 8. This determines whether SpO_2_ readings erroneously change at low signal levels. The full set of results is shown in Appendix 3.

The NDF attenuation tests revealed that the lines are all relatively flat and they do not overestimate SpO_2_ at high attenuation.

### Varying SpO_2_, with synthetic melanin filters

**Fig. 9:**
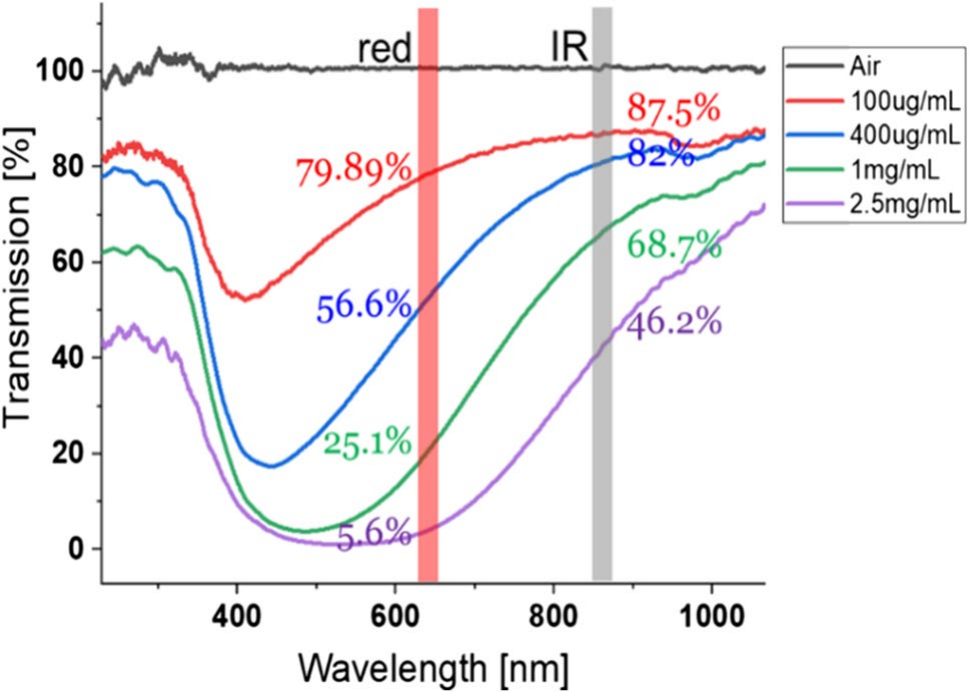
Melanin attenuation spectra as a function of different melanin concentrations demonstrating higher attenuation at red compared to IR wavelengths

The effects of skin colour are simulated using MFs that attenuate red light more than IR. At red, relative attenuation of the transmitted light was 9% (D), 44.8% (F), 173% (I) and 725% (J) greater than at IR. In Fig. 9, the measured transmission spectrum shows this unequal attenuation at red and IR wavelengths. Due to the low intensity of the halogen lamp at wavelengths < 500 nm, the results in Fig. 9 are not reliable in this range, however only 660 nm (red) and 880 nm (IR) wavelengths are used in this study.

**Fig. 10:**
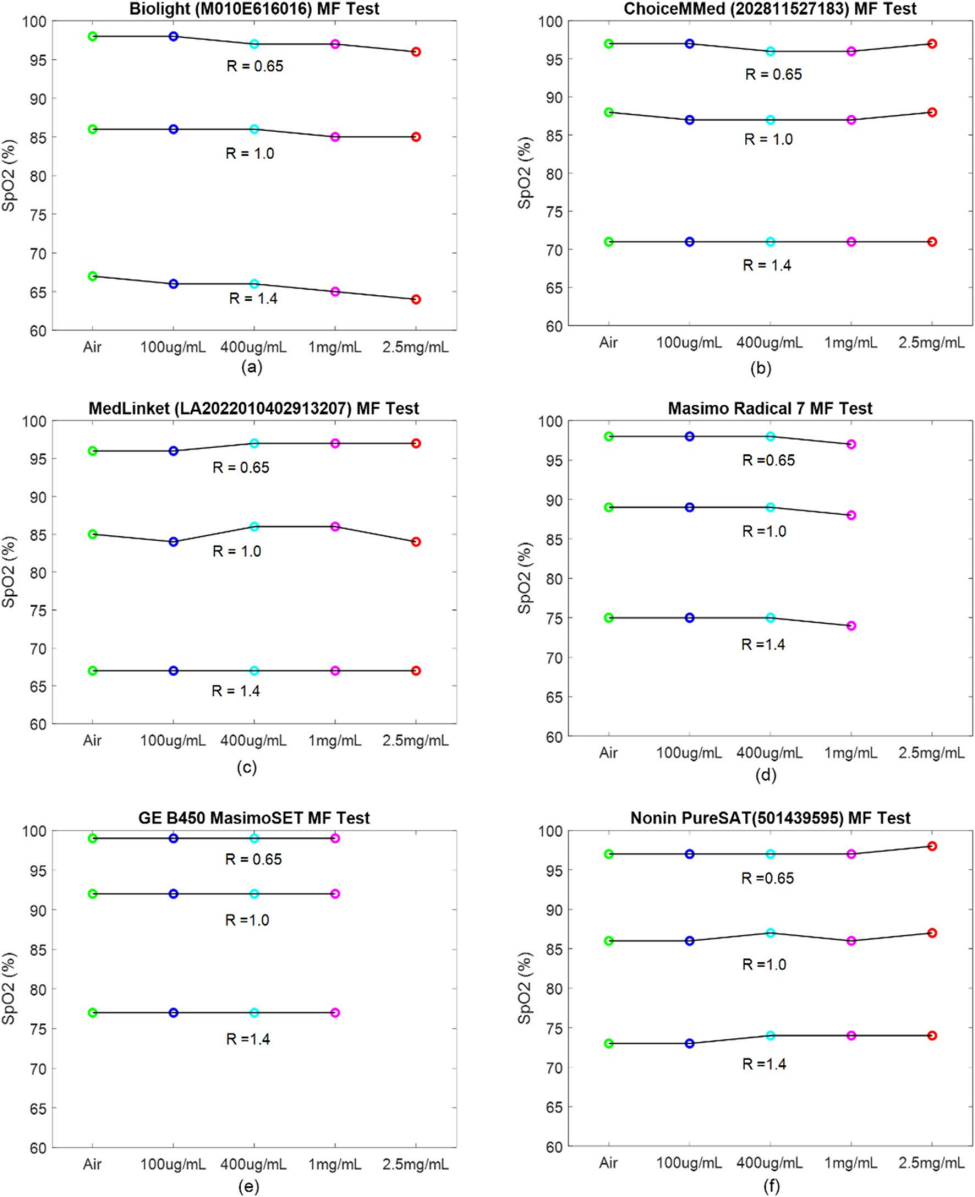
Effect of synthetic melanin filters on different PO devices as a function of R generated by the Whaleteq simulator

The response of each PO under different MFs as a function of R generated by the Whaleteq simulator has been recorded and is shown in Fig. 10. The full set of tests is shown in Appendix 4. Again, similar observations were recorded when the MF was sandwiched between the simulator and the device., The attenuation by melanin is cancelled when calculating R and SpO_2_ is constant with different MFs. The Masimo radical and GE Masimo SET were unable to record a value at the highest melanin attenuation level, rather than reporting an incorrect SpO_2_ value.

## Discussion

In this study, the performance of POs used in the NHS COVID Oximetry @home programme and in hospitals was investigated using a test bench. The test bench enabled the POs to be subjected to low signal and varying melanin attenuation levels across a range of simulated SpO_2_ values under controlled laboratory conditions. The effects of low signal levels were simulated by NDFs that attenuated red and IR light in the same manner while the effects of melanin attenuation were achieved by utilizing MFs that attenuated both red and IR light by unequal amounts. In these tests, the SpO_2_ overestimation, which has often been observed clinically, was not reproduced. Mathematically, if an attenuation α is applied to both red and IR channels equally then α cancels throughout and R (and hence SpO_2_) is not affected, and Eq. (2) becomes the same as Eq. (1).

2 $$R={~}^{\frac{{\mathrm{\alpha }AC}_{red}}{{\mathrm{\alpha }DC}_{red}}}\!\left/ \!{~}_{\frac{{\mathrm{\alpha }AC}_{IR}}{\mathrm{\alpha }{DC}_{IR}}}\right. = {~}^{\frac{{AC}_{red}}{{DC}_{red}}}\!\left/ \!{~}_{\frac{{AC}_{IR}}{{DC}_{IR}}}\right.$$

Melanin provides unequal attenuation of light at red and IR wavelengths. However, this is also compensated mathematically by the PO. If an attenuation α is applied to red and β is applied to IR then there is still cancellation of both α’s in the numerator and β’s in the denominator. *R* (and hence SpO_2_) is theoretically not affected, and Eq. (3) matches Eq. (1).

3 $$R={~}^{\frac{{\mathrm{\alpha }AC}_{red}}{{\mathrm{\alpha }DC}_{red}}}\!\left/ \!{~}_{\frac{{\upbeta AC}_{IR}}{\upbeta {DC}_{IR}}}\right. = {~}^{\frac{{AC}_{red}}{{DC}_{red}}}\!\left/ \!{~}_{\frac{{AC}_{IR}}{{DC}_{IR}}}\right.$$

To produce an incorrect SpO_2_ value, the red and IR wavelength AC and DC components have to be attenuated by different amounts, i.e.,

4 $$R={~}^{\frac{{\mathrm{\alpha }AC}_{red}}{{\upgamma DC}_{red}}}\!\left/ \!{~}_{\frac{{\upbeta AC}_{IR}}{\upphi {DC}_{IR}}}\right.$$

where α, β, γ, ϕ are different attenuation values.

**Fig. 11:**
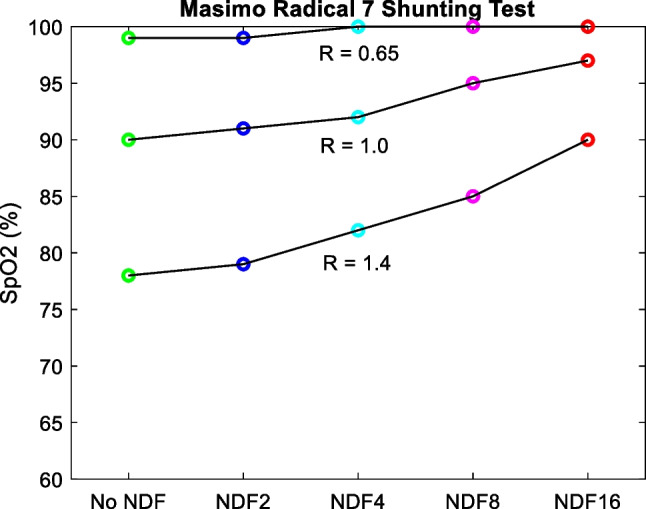
Effect of removing the shunting barrier has a greater effect on red light than IR causing SpO_2_ overestimation due to R decreasing—compared with Fig. 8d which has its shunting barrier intact
  The overall strength of this evaluation presented in this paper is that POs under test were all presented with signals under similar controlled conditions. This removes the limitations of variability over time and between subjects that occur in in vivo studies. For instance, at *R* = 1, the AC and DC intensities were recorded using a spectrometer (Ocean Optics, HDX) over a duration of 20 min (corresponding to the testing period of POs). The measurements revealed variations in intensity < 0.1% for both red and IR wavelengths.
  Avoiding the use of human subjects in device characterisation eliminates physiological variability, errors in experimental protocol, and variations in skin colour, thereby offering an alternative solution to the more complex, time-consuming and invasive ABG procedures. However, not using human subjects is a limitation because a single-layer melanin model oversimplifies optical attenuation due to skin tissue. This model only accounts for the light absorption effect and ignores other critical factors, such as light scattering by tissue and heterogenous distribution of chromophores, that could all contribute to variations in tissue optical behaviour. Moreover, this setup might not accurately mimic the natural modulation of light within the skin tissue where in DC light entering the tissue interacts longer with melanin than the AC component which arises as a result of the pulsatile nature of blood flow. Finally, the relationship between the *R*-ratio and SpO_2_ or any additional processing used in commercial devices is unknown due to its proprietary nature. In the future, the evaluation of POs under bench tests could be improved by either fabricating more realistic skin tissue samples or by pre-programming the Whaleteq to generate signals from numerical simulations such as Monte Carlo that correspond to different skin tones and SpO_2_ levels.

Although these devices performed well according to the basic PO theory, this doesn’t confirm that melanin has no effect on clinically obtained PO readings. Other factors could also influence the performance of a PO at different skin colours, for example, in no particular order:

1. Calibration: when devices are calibrated against ABG analysis for SpO_2_ accuracy there is a requirement to record demographics of subjects. The PO standard (BS EN ISO 80601–2-61:2019) [22] states that, ‘The summary of the clinical study report used to assess SpO_2_ ACCURACY shall state whether the test subjects were sick or healthy and shall describe their skin colour, age and gender.’ However, there is currently no requirement to take skin colour into account in the device design and the relationship between R and SpO_2_. Further, FDA guidelines suggest that clinical trials include at least two dark-pigmented skin or 15% of the population pool whichever is greater for device approval [23]. The current guidelines lack adequate characterisation and representation of dark skin types which is problematic due to the diverse and complex range of skin tones worldwide, potentially leading to increased PO reading bias among non-white skin types. Therefore, we believe that the standard guidelines should incorporate a uniform proportion of light and dark skin types, stratified not subjectively (visual inspection or ethnicity) but by utilizing objective techniques (spectrometer-based) for future device designs.
2. Theory: traditional POs assume that optical path length (OPL) does not change at the red and IR wavelengths within the tissue. However, the OPL within the tissue does change with wavelength and is also dependent on the underlying optical properties [24]. Therefore, OPL terms associated with red and IR will not cancel in the SpO_2_–*R* ratio equation derived from the modified Beer-Lambert law. Moreover, with an increase in melanin concentration, the overall scattering properties of the skin tissue increase [25], resulting in smaller mean free paths and longer OPL. With the increase in wavelength, scattering decreases, as a result, OPL is higher at the red than in the IR wavelength. This could cause apparent substantially low perfusion (due to proportionately small AC generated for a given amount of DC signal) at red than IR resulting in a smaller *R* value—therefore increasing SpO_2_. Further investigation using numerical simulation and basic laboratory phantoms is therefore essential because melanin-induced variations in path length may cause errors due to deviations from the assumptions made in the derivation of the original theory.
3. Instrument design: in our tests, it was possible to create an overestimation of SpO_2_ via direct coupling of light from the source to the detector (i.e., a shunting effect) [26]. Although this is more prevalent in reflection geometry PO, inadequate conformity of the finger to the device could allow this artefact to be present and is worth further investigation. To demonstrate this, it is important to note that the setup shown in Fig. 3 has been designed with a barrier to prevent direct coupling of light between the source and detector. To simulate the effect of shunting this barrier was removed to allow light to leak from source to detector which allowed different DC attenuation levels with wavelength as shown in Eq. (4). Figure 11 (which should be compared to Fig. 8d) shows that conditions for overestimation of SpO_2_ can be created when there is unequal wavelength attenuation of reflected DC light components.

## Conclusion

A PO test bench has been developed to investigate the effects of low signal-to-noise ratio and melanin on a range of simulated SpO_2_ values under controlled laboratory conditions. The POs under test are utilised in the NHS COVID Oximetry @home programme or are commonly used in hospital-based care. All POs demonstrated that the ratio of ratios is effective at cancelling the effects of red and IR being attenuated equally (via NDF) and unequally (via MF). For very high attenuation (low signal-to-noise ratio) the POs do not provide a readout, rather than overestimate SpO_2_. Independent of attenuation, different POs demonstrate the inverse relationship between *R*-ratio and SpO_2_. However, there are large differences between different brands of POs at low (< 80%) oxygen saturations, which is likely due to a paucity of low SpO_2_ calibration data.

These controlled laboratory tests do not conclusively demonstrate that melanin does not affect SpO_2_ measurements, so we need to be cautious not to overinterpret these results. Several peer-reviewed clinical studies have demonstrated an overestimation of SpO_2_ in the presence of higher melanin concentration and so further investigation is necessary in the areas of (i) calibration; (ii) theory and (iii) instrument design.

## Supplementary Information

Below is the link to the electronic supplementary material.

Supplementary file1 (DOCX 4629 KB)

## Data Availability

All data generated during this study are available upon reasonable request by contacting the corresponding author at eezspm@exmail.nottingham.ac.uk.
