# Correction to: Pulse oximeter bench tests under different simulated skin tones

**DOI:** 10.1007/s11517-025-03511-x

**Published:** 2026-01-13

**Authors:** Suvvi K Narayana Swamy, Chenyang He, Barrie R. Hayes‑Gill, Daniel J. Clark, Sarah Green, Stephen Morgan

**Affiliations:** 1 Optics and Photonics Research Group and Centrefor Healthcare Technologies, University of Nottingham, University Park, Nottingham, UK; 2 Clinical Engineering Department, Nottingham UniversityHospitals NHS Trust, Nottingham, UK

**Correction to: Medical & Biological Engineering & Computing**

10.1007/s11517-024-03091-2

The original version of this article unfortunately contained mistakes. A reader alerted us to an issue in Table 3. We confirm that the **ITA°** value for the **2.5 mg/mL (J)** melanin filter is **+ 69.17°,** as originally reported. However, the corresponding b* entry was typeset **without its minus sign.** The correct value is b* = −13.98.

At first glance this row can appear internally inconsistent. As noted in the final paragraph of Sect. 2.3.2, transmission spectral measurement (Fig. 9) was acquired in a **low signal-to-noise **regime, reducing the blue–green contrast and yielding a negative b* for this slide. In other words, the **ITA° value is correct for the measured data,** and the issue was the missing minus sign on b*. We also note that although the computed **ITA° **classifies this entry as “very light,” the L* for the same row is **very low** (dark appearance); this discrepancy reflects the low-SNR conditions rather than a limitation of the ITA method. For clarity, the **sign of ITA°** follows the sign of $\:({L}^{*}-50)/{b}^{*}$.

This correction **does not change** the results, figures, or conclusions of the article.

The original article has been corrected.

