# Supplementary material for: Pulse oximeter bench tests under different simulated skin tones
Source: Med Biol Eng Comput. 2024 Apr 24;63(7):1931–42. doi: 10.1007/s11517-024-03091-2 (PMC12204950; doi:10.1007/s11517-024-03091-2)
Supplement: Supplementary file 1 — Supplementary file1 (DOCX 4629 KB) [file 11517_2024_3091_MOESM1_ESM.docx]

**Appendix 1**

This section displays full set of photographs of the different types of pulse oximeters investigated.


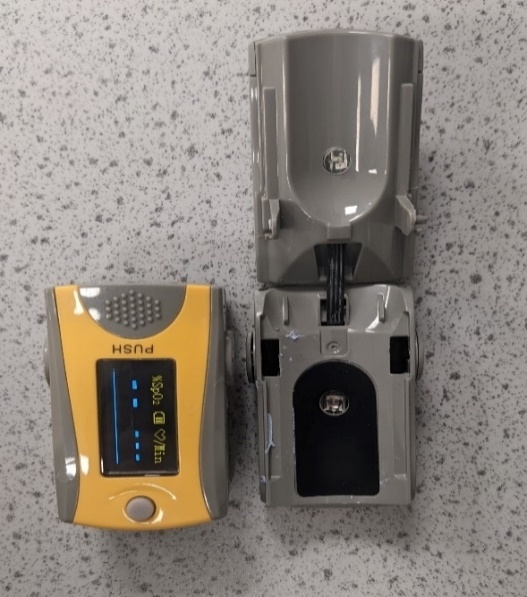

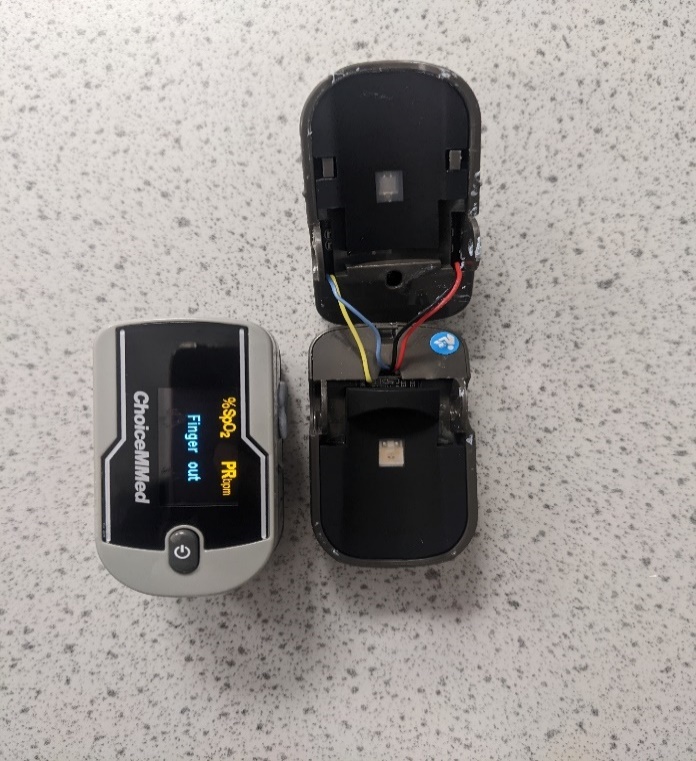


**a. Biolight b. ChoiceMMed A**


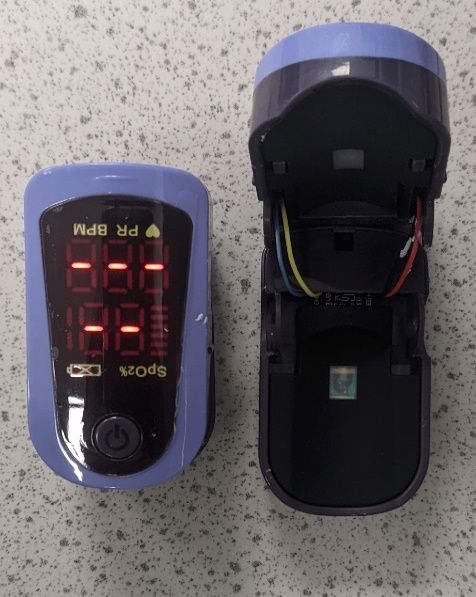

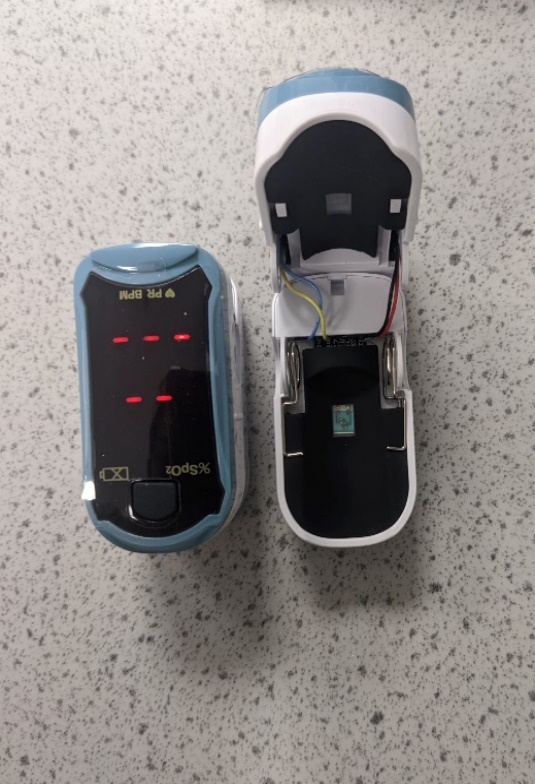


**c. ChoiceMMed B d. ChoiceMMed C**


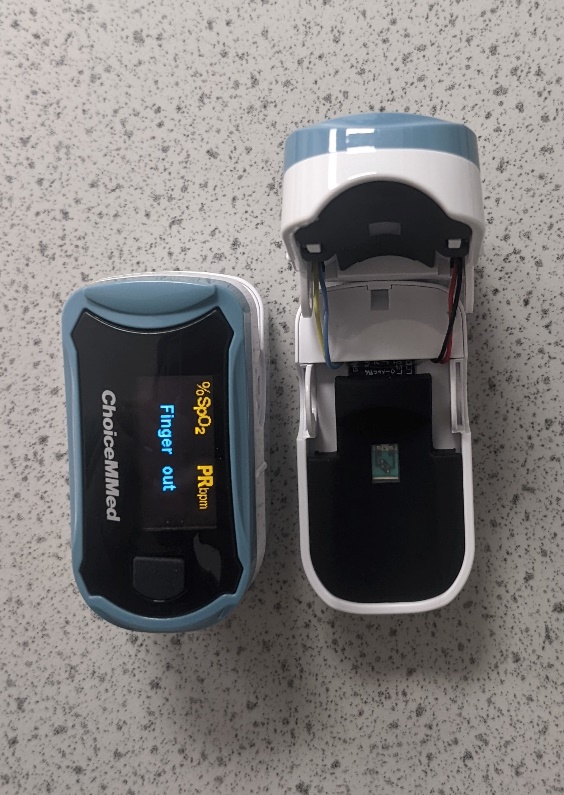

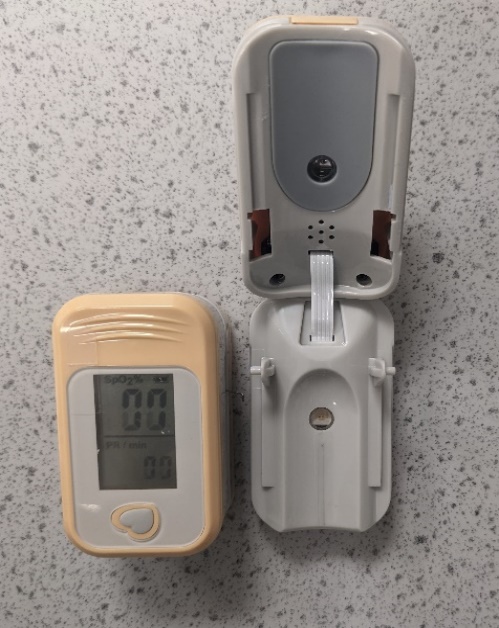


**e. ChoiceMMed D f. MedLinket**

**g. Masimo LNCS Neo L h. Nonin PureSAT**


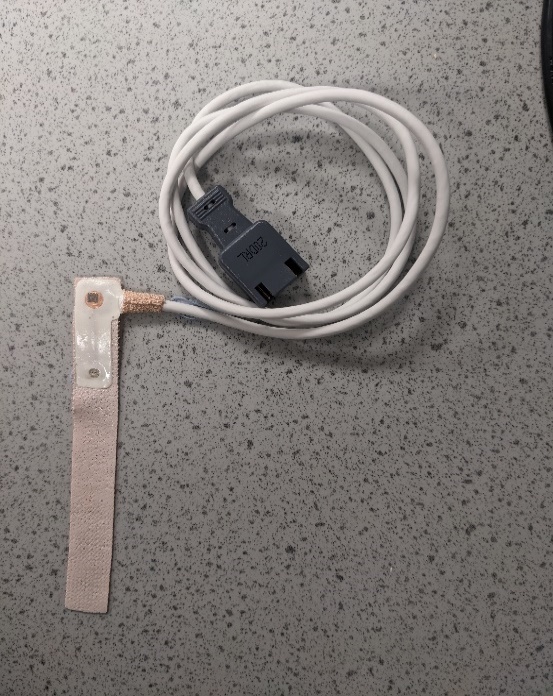

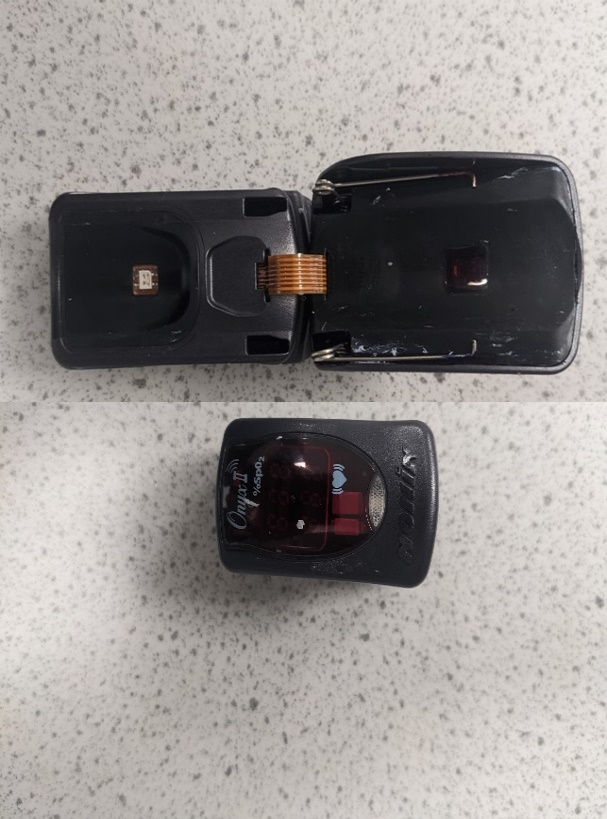


**Figure 12:** Photographs of the pulse oximeter sensors investigated in this work. The Masimo LNCS Neo L sensor is used in conjunction with both the Masimo Radical 7 and the GE B450 Masimo SET

**Appendix 2 – CIE LAB characterization of Melanin filters**

For objective colour quantification, CIE LAB technique [Reference #20, #21 in the manuscript] was utilised and filters were stratified by following the steps 1-3, in order to estimate L* (indicates lightness/darkness), a* (red/green), b* (yellow/blue) and ITA angles for different melanin filters.

Step 1: Obtain tristimulus XYZ values for the CIE 10-degree standard observer and D65 standard illuminant.

X = 0.94, Y = 1, and Z = 1.07

Step 2: XYZ values for different filters are estimated by multiplying the transmission spectrum with D65 standard illuminant and 10-degree observer function spectrums.

Table 4: Shows tristimulus XYZ values for the samples.

| Filters | X_s | Y_s | Z_s |
| --- | --- | --- | --- |
| 100ug/mL | 0.66 | 0.68 | 0.60 |
| 400ug/mL | 0.35 | 0.33 | 0.20 |
| 1mg/mL | 0.10 | 0.81 | 0.57 |
| 2.5mg/mL | 0.025 | 0.016 | 0.035 |

Step 3: L*a*b* values and ITA angles were calculated using the equations below:

L* = $116 {( \frac{Y}{Y_{\_s}} )}^{1/3}$ – 16

a* = $500[ {( \frac{X}{X\_s} )}^{1/3}$ – ${( \frac{Y}{Y\_s} )}^{1/3}$]

b* = $200[ {( \frac{Y}{Y\_s} )}^{1/3}$ – ${( \frac{Z}{Z\_s} )}^{1/3}$]

ITA = [arctan (L* - 50) / b*] ×180/π

The estimated L*, a*, b*, ITA angles and their corresponding skin colour (according to the ITA chart) are summarised in Table 3 in the main body of the paper.

**Appendix 3 – Neutral density filter results**

This section displays the full set of test results recorded when pulse oximeters were presented with different neutral density filters (NDF) as a function of R generated by the Whaleteq simulator.

1. **Bio-light neutral density filter (NDF) test with R generated by the Whaleteq simulator.**

Figure 13. Effect of attenuating red and infrared light equally using NDFs on Biolight pulse oximeters as a function of R generated by the Whaleteq simulator.

**2) ChoiceMMed A neutral density filter (NDF) test with R generated by the Whaleteq simulator.**

Figure 14. Effect of attenuating red and infrared light equally using NDFs on ChoiceMMed A pulse oximeters as a function of R generated by the Whaleteq simulator.

1. **ChoiceMMed B neutral density filter (NDF) test with R generated by the Whaleteq simulator**

Figure 15. Effect of attenuating red and infrared light equally using NDFs on ChoiceMMed B pulse oximeters as a function of R generated by the Whaleteq simulator.

**4) ChoiceMMed C neutral density filter (NDF) test with R generated by the Whaleteq simulator.**

Figure 16. Effect of attenuating red and infrared light equally using NDFs on ChoiceMMed C pulse oximeters vs NDF as a function of R generated by the Whaleteq simulator.

**5) ChoiceMMed D neutral density filter (NDF) test with R generated by the Whaleteq simulator.**

Figure 17. Effect of attenuating red and infrared light equally using NDFs on ChoiceMMed D pulse oximeters as a function of R generated by the Whaleteq simulator.

**6) MedLinket neutral density filter (NDF) test with R generated by the Whaleteq simulator.**

Figure 18. Effect of attenuating red and infrared light equally using NDFs on MedLinket pulse oximeters as a function of R generated by the Whaleteq simulator.

**Appendix 4 – Synthetic melanin filter results**

This section displays full set of test results recorded when pulse oximeters were presented with different melanin filters with R generated by the Whaleteq simulator as a parameter.

1. **Biolight synthetic melanin filter (MF) test with R generated by the Whaleteq simulator.**

Figure 19. Effect of synthetic melanin filters on Biolight pulse oximeters as a function of Whaleteq simulator R value.

1. **ChoiceMMed synthetic melanin filter (MF) test with R generated by the Whaleteq simulator.**

Figure 20. Effect of synthetic melanin filters on ChoiceMMed pulse oximeters as a function of Whaleteq simulator R value.

1. **MedLinket synthetic melanin filter (MF) test with R generated by the Whaleteq simulator.**

Figure 21. Effect of synthetic melanin filters on MedLinket pulse oximeters as a function of Whaleteq simulator R value.
